# Acrylato[tris­(1-methyl-1*H*-benzimidazol-2-ylmeth­yl)amine]cobalt(II) perchlorate–dimethyl­formamide–methanol (2/2/3)

**DOI:** 10.1107/S1600536808032595

**Published:** 2008-10-15

**Authors:** Huilu Wu, Wei Ying, Kaitong Wang, Xingcai Huang, Ke Li

**Affiliations:** a School of Chemical and Biological Engineering, Lanzhou Jiaotong University, Lanzhou 730070, People’s Republic of China; b College of Chemistry and Chemical Engineering, Lanzhou University, Lanzhou 730000, People’s Republic of China

## Abstract

In the title complex, [Co(C_3_H_3_O_2_)(C_27_H_27_N_7_)]ClO_4_·C_3_H_7_NO·1.5CH_4_O, the Co^II^ ion is five-coordinated by four N atoms from a tris­(1-methyl-1*H*-benzimidazol-2-ylmeth­yl)amine (mentb) ligand and one O atom from an acrylate ligand in a distorted trigonal–bipyramidal geometry with approximate mol­ecular *C*_3_ symmetry. The atoms of the acrylate ligand are disordered over two sites, with approximate occupancies of 0.90 and 0.10. In addition, the solvent hemimethanol mol­ecule is disordered over two positions with equal occupancies. The crystal structure is stabilized by weak intermolecular O—H⋯O hydrogen bonds.

## Related literature

For background information, see: Youngme *et al.* (2007 ▶). For bond-length data, see: Allen *et al.* (1987 ▶).
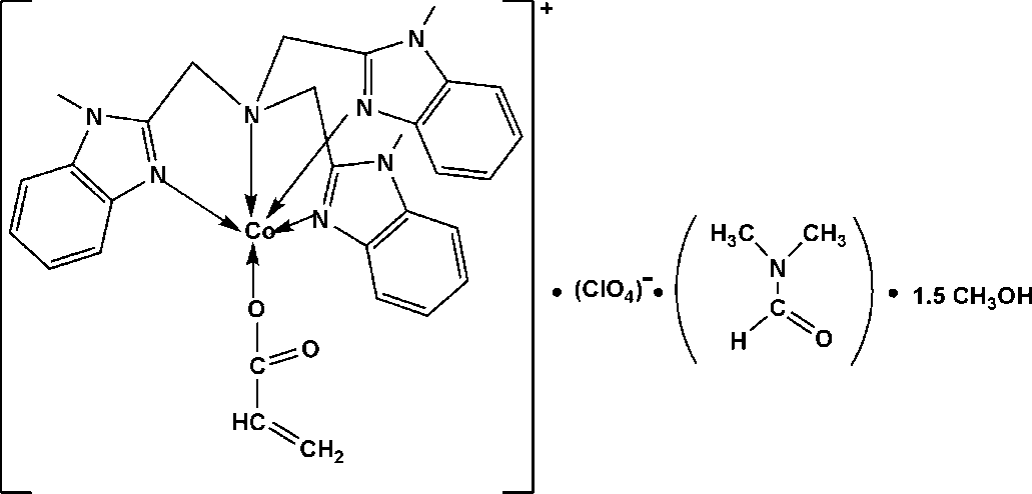


## Experimental

### 

#### Crystal data

[Co(C_3_H_3_O_2_)(C_27_H_27_N_7_)]ClO_4_·C_3_H_7_NO·1.5CH_4_O
*M* *_r_* = 800.15
Triclinic, 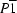

*a* = 11.3398 (3) Å
*b* = 13.9507 (4) Å
*c* = 14.4270 (5) Å
α = 108.443 (1)°
β = 110.738 (1)°
γ = 100.278 (1)°
*V* = 1913.24 (10) Å^3^
*Z* = 2
Mo *K*α radiation
μ = 0.58 mm^−1^
*T* = 153 (2) K
0.35 × 0.32 × 0.26 mm

#### Data collection

Rigaku R-AXIS Spider diffractometer
Absorption correction: multi-scan (*ABSCOR*; Higashi, 1995 ▶) *T*_min_ = 0.823, *T*_max_ = 0.864
15711 measured reflections
7032 independent reflections
6085 reflections with *I* > 2σ(*I*)
*R*_int_ = 0.020

#### Refinement

*R*[*F* ^2^ > 2σ(*F* ^2^)] = 0.046
*wR*(*F* ^2^) = 0.147
*S* = 1.07
7032 reflections
519 parameters
6 restraints
H-atom parameters constrained
Δρ_max_ = 1.26 e Å^−3^
Δρ_min_ = −0.44 e Å^−3^

### 

Data collection: *RAPID-AUTO* (Rigaku/MSC, 2004 ▶); cell refinement: *RAPID-AUTO*; data reduction: *RAPID-AUTO*; program(s) used to solve structure: *SHELXS97* (Sheldrick, 2008 ▶); program(s) used to refine structure: *SHELXL97* (Sheldrick, 2008 ▶); molecular graphics: *SHELXTL* (Sheldrick, 2008 ▶); software used to prepare material for publication: *SHELXTL* and *PLATON* (Spek, 2003 ▶).

## Supplementary Material

Crystal structure: contains datablocks global, I. DOI: 10.1107/S1600536808032595/lh2706sup1.cif

Structure factors: contains datablocks I. DOI: 10.1107/S1600536808032595/lh2706Isup2.hkl

Additional supplementary materials:  crystallographic information; 3D view; checkCIF report

## Figures and Tables

**Table 1:** Hydrogen-bond geometry (Å, °)

*D*—H⋯*A*	*D*—H	H⋯*A*	*D*⋯*A*	*D*—H⋯*A*
O8—H8O⋯O7^i^	0.84	1.94	2.765 (4)	169

Symmetry code: (i) 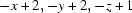.

**Fig. 1.**
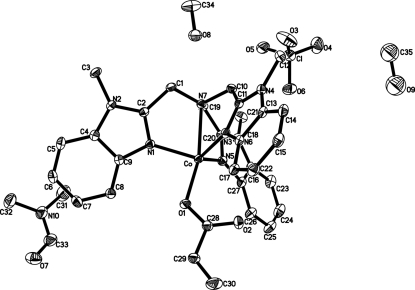
Molecular structure and atom numbering for the components of (I). Hydrogen atoms have been omitted for clarity and the displacement ellipsoids are shown at the 30% probability level.

**Fig. 2.**
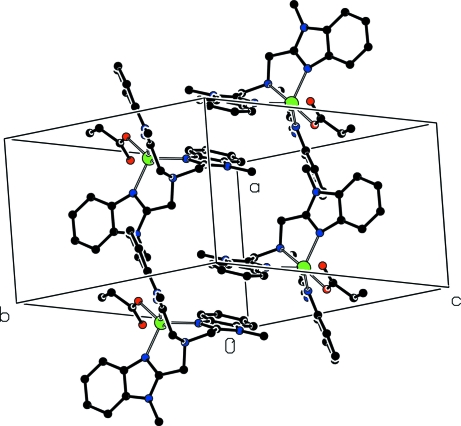
The packing of the cations. Neither the disorder or H atoms are shown.

## supplementary crystallographic information

### Comment

The asymmetric unit of the title compound (I), (Fig. 1), consists of a discrete [Co(mentb)(acrylate)] cation, a perchlorate anion, a DMF molecule and 1.5 molecules of methanol. The cobalt ion is five-coordinate with a N_4_O ligand set. The mentb ligand acts as a tetradentate N-donor, and an O atom of the carboxylate group of the acrylate ligand completes the coordination. The coordination geometry of the Co^II^ ion may be best described as distorted trigonal bipyramidal (τ = 0.87), with approximate site symmetry C_3_. The parameter τ is defined as (β - α)/60 [where β = O1—Co—N7, α = N3—Co—N5] and its value varies from 0 (in regular square-based pyramidal) to 1 (in regular trigonal bipyramidal) [Youngme *et al.*, 2007]. This geometry is assumed by the Co^II^ ion presumably to relieve the steric crowding. The equatorial plane is occupied by three N atoms of three benzimidazolyl groups, while the Co^II^ ion protrudes towards atom O1 and is 0.528 (2) Å from the plane of atoms N1/N3/N5. The axial positions are occupied by atoms N7 and O1. The three benzimidazole ring arms of the mentb ligand form a cone-shaped cavity. The distance between Co^II^ and O2 is 3.076 (2) A, so atom O2 is not considered to be coordinated. The angles and distances in the mentb and salicylate are normally equal [for standard bond lengths, see: Allen *et al.*, 1987]. The crystal structure is stabilized by weak intermolecular O-H···O hydrogen bonds and weak π···π stacking interactions (Fig. 2). The significant stacking interactions have ring centroid···ring centroid distances in the range 3.456 (2)-3.646 (2)Å.

### Experimental

To a stirred solution of tris(1-methyl-1H-benzimidazol-2-ylmethyl)amine (0.0899 g, 0.2 mmol) in hot MeOH (10 ml) was added Co(ClO_4_)_2_ (H_2_O)_6_ (0.0732 g, 0.2 mmol), followed by a solution of Na(acrylate) (0.0188 g, 0.2 mmol) in MeOH (5 ml). A red crystalline product formed rapidly. The precipitate was filtered off, washed with MeOH and absolute Et_2_O, and dried *in vacuo*. The dried precipitate was dissolved in DMF to a red solution that was allowed to evaporate at room temperature. The red crystals suitable for X-ray diffraction studies were obtained after four weeks. Yield, 0.106 g (66%). (found: C, 51.67; H, 5.38; N,14.21. Calcd. for C34.50H43ClN8O8.50Co: C, 51.79; H, 5.42; N, 14.00)

### Refinement

All H atoms were found in difference electron maps and were subsequently refined in a riding-model approximation with C—H distances ranging from 0.95 to 0.99 Å and O—H distance 0.84 Å and *U*_iso_(H) = 1.2 *U*_eq_ of the carrier atom,

### Crystal data

**Table:**

[Co(C_3_H_3_O_2_)(C_27_H_27_N_7_)]ClO_4_·C_3_H_7_NO·1.5CH_4_O	*Z* = 2
*M**_r_* = 800.15	*F*(000) = 836
Triclinic, *P*1	*D*_x_ = 1.389 Mg m^−^^3^
Hall symbol: -P 1	Mo *K*α radiation, λ = 0.71073 Å
*a* = 11.3398 (3) Å	Cell parameters from 7032 reflections
*b* = 13.9507 (4) Å	θ = 3.0–25.5°
*c* = 14.4270 (5) Å	µ = 0.58 mm^−^^1^
α = 108.443 (1)°	*T* = 153 K
β = 110.738 (1)°	Block, red
γ = 100.278 (1)°	0.35 × 0.32 × 0.26 mm
*V* = 1913.24 (10) Å^3^	

### Data collection

**Table:**

Rigaku R-AXIS Spider diffractometer	7032 independent reflections
Radiation source: Rotating Anode	6085 reflections with *I* > 2σ(*I*)
graphite	*R*_int_ = 0.020
ω scans	θ_max_ = 25.5°, θ_min_ = 3.0°
Absorption correction: multi-scan (ABSCOR; Higashi, 1995)	*h* = −13→13
*T*_min_ = 0.823, *T*_max_ = 0.864	*k* = −15→16
15711 measured reflections	*l* = −17→17

### Refinement

**Table:**

Refinement on *F*^2^	Primary atom site location: structure-invariant direct methods
Least-squares matrix: full	Secondary atom site location: difference Fourier map
*R*[*F*^2^ > 2σ(*F*^2^)] = 0.046	Hydrogen site location: inferred from neighbouring sites
*wR*(*F*^2^) = 0.147	H-atom parameters constrained
*S* = 1.07	*w* = 1/[σ^2^(*F*_o_^2^) + (0.0954*P*)^2^ + 1.1776*P*] where *P* = (*F*_o_^2^ + 2*F*_c_^2^)/3
7032 reflections	(Δ/σ)_max_ = 0.001
519 parameters	Δρ_max_ = 1.26 e Å^−^^3^
6 restraints	Δρ_min_ = −0.44 e Å^−^^3^

### Special details

**Table:**

Geometry. All e.s.d.'s (except the e.s.d. in the dihedral angle between two l.s. planes) are estimated using the full covariance matrix. The cell e.s.d.'s are taken into account individually in the estimation of e.s.d.'s in distances, angles and torsion angles; correlations between e.s.d.'s in cell parameters are only used when they are defined by crystal symmetry. An approximate (isotropic) treatment of cell e.s.d.'s is used for estimating e.s.d.'s involving l.s. planes.
Refinement. Refinement of *F*^2^ against ALL reflections. The weighted *R*-factor *wR* and goodness of fit *S* are based on *F*^2^, conventional *R*-factors *R* are based on *F*, with *F* set to zero for negative *F*^2^. The threshold expression of *F*^2^ > σ(*F*^2^) is used only for calculating *R*-factors(gt) *etc*. and is not relevant to the choice of reflections for refinement. *R*-factors based on *F*^2^ are statistically about twice as large as those based on *F*, and *R*- factors based on ALL data will be even larger.

### Fractional atomic coordinates and isotropic or equivalent isotropic displacement parameters (Å^2^)

**Table:**

	*x*	*y*	*z*	*U*_iso_*/*U*_eq_	Occ. (<1)
Co	0.85373 (3)	0.66082 (3)	0.32233 (3)	0.02259 (13)	
Cl	0.39694 (7)	0.32230 (6)	0.32447 (6)	0.03835 (19)	
O1	0.9876 (2)	0.71250 (18)	0.27486 (15)	0.0330 (7)	0.901 (5)
O2	0.9039 (2)	0.56682 (14)	0.12161 (16)	0.0361 (7)	0.901 (5)
O1'	0.932 (2)	0.6236 (16)	0.2363 (16)	0.044 (7)	0.099 (5)
O2'	0.986 (3)	0.7549 (7)	0.218 (3)	0.13 (2)	0.099 (5)
O3	0.3275 (3)	0.3884 (3)	0.2891 (3)	0.0793 (10)	
O4	0.3064 (3)	0.2197 (2)	0.2890 (2)	0.0725 (8)	
O5	0.4629 (2)	0.3703 (2)	0.4417 (2)	0.0591 (7)	
O6	0.4972 (2)	0.3160 (2)	0.28583 (19)	0.0499 (6)	
O7	1.6359 (3)	1.0565 (2)	0.7244 (3)	0.0626 (7)	
O8	0.4466 (2)	0.78673 (17)	0.33218 (19)	0.0411 (5)	
H8O	0.4266	0.8330	0.3098	0.049*	
N1	0.9381 (2)	0.79702 (17)	0.46404 (16)	0.0234 (4)	
N2	0.9256 (2)	0.91605 (17)	0.60230 (17)	0.0261 (5)	
N3	0.6689 (2)	0.64681 (16)	0.21381 (16)	0.0240 (4)	
N4	0.4473 (2)	0.58321 (17)	0.14350 (17)	0.0259 (5)	
N5	0.8638 (2)	0.52411 (17)	0.34938 (17)	0.0263 (5)	
N6	0.8178 (2)	0.41123 (19)	0.42099 (18)	0.0301 (5)	
N7	0.7151 (2)	0.64080 (17)	0.40828 (17)	0.0238 (4)	
N10	1.5434 (3)	1.0109 (2)	0.8268 (3)	0.0549 (8)	
C1	0.7256 (2)	0.7501 (2)	0.4742 (2)	0.0272 (5)	
H1A	0.7061	0.7509	0.5360	0.033*	
H1B	0.6607	0.7750	0.4293	0.033*	
C2	0.8638 (2)	0.8210 (2)	0.5149 (2)	0.0241 (5)	
C3	0.8707 (3)	0.9685 (2)	0.6741 (2)	0.0366 (7)	
H3A	0.7731	0.9378	0.6375	0.044*	
H3B	0.8970	1.0453	0.6917	0.044*	
H3C	0.9051	0.9577	0.7413	0.044*	
C4	1.0501 (3)	0.9576 (2)	0.6089 (2)	0.0269 (5)	
C5	1.1554 (3)	1.0522 (2)	0.6819 (2)	0.0345 (6)	
H5	1.1500	1.1030	0.7409	0.041*	
C6	1.2672 (3)	1.0684 (2)	0.6645 (2)	0.0373 (7)	
H6	1.3411	1.1318	0.7131	0.045*	
C7	1.2759 (3)	0.9943 (2)	0.5770 (3)	0.0360 (6)	
H7	1.3548	1.0088	0.5674	0.043*	
C8	1.1712 (3)	0.9002 (2)	0.5046 (2)	0.0283 (6)	
H8	1.1770	0.8496	0.4456	0.034*	
C9	1.0573 (2)	0.8826 (2)	0.5214 (2)	0.0238 (5)	
C10	0.5791 (2)	0.5790 (2)	0.3218 (2)	0.0260 (5)	
H10A	0.5129	0.5983	0.3475	0.031*	
H10B	0.5637	0.5015	0.3013	0.031*	
C11	0.5648 (2)	0.60425 (19)	0.2267 (2)	0.0240 (5)	
C12	0.3149 (3)	0.5384 (2)	0.1345 (2)	0.0318 (6)	
H12A	0.3172	0.4867	0.1673	0.038*	
H12B	0.2503	0.5025	0.0576	0.038*	
H12C	0.2885	0.5960	0.1726	0.038*	
C13	0.4752 (3)	0.61622 (19)	0.0702 (2)	0.0256 (5)	
C14	0.3922 (3)	0.6132 (2)	−0.0293 (2)	0.0308 (6)	
H14	0.2978	0.5849	−0.0592	0.037*	
C15	0.4537 (3)	0.6533 (2)	−0.0823 (2)	0.0334 (6)	
H15	0.4005	0.6532	−0.1501	0.040*	
C16	0.5937 (3)	0.6943 (2)	−0.0384 (2)	0.0332 (6)	
H16	0.6326	0.7216	−0.0772	0.040*	
C17	0.6760 (3)	0.6959 (2)	0.0598 (2)	0.0296 (6)	
H17	0.7704	0.7231	0.0887	0.036*	
C18	0.6152 (2)	0.65610 (19)	0.11455 (19)	0.0233 (5)	
C19	0.7642 (3)	0.5831 (2)	0.4753 (2)	0.0283 (6)	
H19A	0.6911	0.5449	0.4859	0.034*	
H19B	0.8366	0.6338	0.5475	0.034*	
C20	0.8152 (2)	0.5053 (2)	0.4155 (2)	0.0263 (5)	
C21	0.7751 (3)	0.3659 (3)	0.4873 (3)	0.0378 (7)	
H21A	0.7347	0.4119	0.5237	0.045*	
H21B	0.8526	0.3613	0.5420	0.045*	
H21C	0.7096	0.2942	0.4404	0.045*	
C22	0.8701 (3)	0.3637 (2)	0.3524 (2)	0.0312 (6)	
C23	0.8903 (3)	0.2653 (2)	0.3241 (2)	0.0393 (7)	
H23	0.8661	0.2158	0.3521	0.047*	
C24	0.9470 (3)	0.2433 (3)	0.2537 (3)	0.0450 (8)	
H24	0.9626	0.1768	0.2324	0.054*	
C25	0.9826 (3)	0.3152 (3)	0.2123 (2)	0.0429 (8)	
H25	1.0239	0.2975	0.1652	0.051*	
C26	0.9593 (3)	0.4119 (2)	0.2381 (2)	0.0342 (6)	
H26	0.9823	0.4604	0.2087	0.041*	
C27	0.9011 (3)	0.4354 (2)	0.3086 (2)	0.0283 (6)	
C28	0.9799 (2)	0.65979 (16)	0.18204 (15)	0.0300 (6)	
C29	1.0710 (3)	0.7152 (3)	0.1482 (3)	0.0325 (8)	0.901 (5)
H29	1.1327	0.7843	0.1984	0.039*	0.901 (5)
C29'	1.036 (4)	0.643 (5)	0.120 (4)	0.055 (11)	0.099 (5)
H29'	1.0652	0.5833	0.1212	0.066*	0.099 (5)
C30	1.0707 (4)	0.6743 (3)	0.0539 (3)	0.0543 (9)	
H30A	1.0100	0.6053	0.0021	0.065*	
H30B	1.1311	0.7133	0.0366	0.065*	
C31	1.4675 (7)	0.9251 (4)	0.8413 (5)	0.102 (2)	
H31A	1.4460	0.8564	0.7820	0.122*	
H31B	1.3847	0.9373	0.8404	0.122*	
H31C	1.5208	0.9242	0.9110	0.122*	
C32	1.5733 (5)	1.1192 (3)	0.9011 (4)	0.0640 (11)	
H32A	1.6163	1.1692	0.8788	0.077*	
H32B	1.6334	1.1307	0.9748	0.077*	
H32C	1.4904	1.1312	0.9002	0.077*	
C33	1.5787 (4)	0.9898 (3)	0.7467 (3)	0.0533 (9)	
H33	1.5583	0.9167	0.7023	0.064*	
C34	0.3940 (4)	0.7816 (4)	0.4049 (4)	0.0641 (11)	
H34A	0.4298	0.7369	0.4403	0.077*	
H34B	0.2966	0.7505	0.3654	0.077*	
H34C	0.4186	0.8538	0.4600	0.077*	
C35	0.103 (2)	0.0960 (19)	0.008 (2)	0.097 (5)*	0.25
H35A	0.1537	0.1582	0.0783	0.116*	0.25
H35B	0.0371	0.0450	0.0143	0.116*	0.25
H35C	0.0562	0.1191	−0.0477	0.116*	0.25
O9	0.192 (2)	0.0453 (16)	−0.0210 (17)	0.119 (6)*	0.25
H9O	0.1654	−0.0198	−0.0342	0.143*	0.25
O9'	0.0197 (16)	−0.0281 (13)	−0.0351 (13)	0.103 (5)*	0.25
H9O'	−0.0390	−0.0076	−0.0706	0.123*	0.25
C35'	0.1498 (19)	0.0451 (19)	0.001 (2)	0.097 (5)*	0.25
H35D	0.1991	0.0079	−0.0330	0.116*	0.25
H35E	0.1986	0.0723	0.0804	0.116*	0.25
H35F	0.1403	0.1049	−0.0192	0.116*	0.25

### Atomic displacement parameters (Å^2^)

**Table:**

	*U*^11^	*U*^22^	*U*^33^	*U*^12^	*U*^13^	*U*^23^
Co	0.0241 (2)	0.0256 (2)	0.01876 (19)	0.00885 (15)	0.01064 (14)	0.00814 (15)
Cl	0.0424 (4)	0.0413 (4)	0.0393 (4)	0.0115 (3)	0.0212 (3)	0.0237 (3)
O1	0.0313 (11)	0.0420 (16)	0.0238 (12)	0.0071 (10)	0.0163 (9)	0.0093 (10)
O2	0.0368 (12)	0.0301 (12)	0.0278 (12)	0.0009 (10)	0.0079 (9)	0.0081 (10)
O1'	0.047 (13)	0.025 (13)	0.070 (17)	0.016 (10)	0.028 (12)	0.028 (12)
O2'	0.041 (16)	0.22 (5)	0.034 (18)	−0.03 (2)	0.002 (13)	0.00 (3)
O3	0.0489 (15)	0.098 (2)	0.131 (3)	0.0352 (16)	0.0395 (17)	0.089 (2)
O4	0.093 (2)	0.0444 (15)	0.0691 (19)	−0.0045 (14)	0.0426 (17)	0.0164 (13)
O5	0.0536 (14)	0.0789 (18)	0.0389 (13)	0.0181 (13)	0.0253 (11)	0.0135 (13)
O6	0.0583 (14)	0.0663 (16)	0.0415 (13)	0.0240 (12)	0.0330 (11)	0.0274 (12)
O7	0.0648 (16)	0.0669 (17)	0.093 (2)	0.0363 (14)	0.0515 (16)	0.0508 (16)
O8	0.0404 (11)	0.0401 (12)	0.0503 (13)	0.0156 (10)	0.0258 (10)	0.0196 (10)
N1	0.0243 (10)	0.0256 (11)	0.0207 (10)	0.0088 (9)	0.0105 (8)	0.0091 (9)
N2	0.0313 (11)	0.0252 (11)	0.0237 (11)	0.0114 (9)	0.0145 (9)	0.0085 (9)
N3	0.0266 (10)	0.0237 (11)	0.0188 (10)	0.0068 (9)	0.0090 (8)	0.0072 (8)
N4	0.0244 (10)	0.0252 (11)	0.0228 (11)	0.0064 (9)	0.0084 (9)	0.0066 (9)
N5	0.0277 (11)	0.0273 (11)	0.0240 (11)	0.0110 (9)	0.0108 (9)	0.0102 (9)
N6	0.0313 (11)	0.0347 (12)	0.0279 (12)	0.0128 (10)	0.0110 (9)	0.0184 (10)
N7	0.0230 (10)	0.0272 (11)	0.0215 (10)	0.0085 (9)	0.0101 (8)	0.0096 (9)
N10	0.069 (2)	0.0374 (15)	0.062 (2)	0.0160 (14)	0.0381 (17)	0.0153 (14)
C1	0.0267 (12)	0.0308 (14)	0.0262 (13)	0.0126 (11)	0.0151 (11)	0.0089 (11)
C2	0.0267 (12)	0.0269 (13)	0.0214 (12)	0.0109 (10)	0.0113 (10)	0.0112 (10)
C3	0.0473 (17)	0.0342 (15)	0.0338 (15)	0.0167 (13)	0.0264 (13)	0.0090 (12)
C4	0.0316 (13)	0.0245 (13)	0.0268 (13)	0.0109 (11)	0.0117 (11)	0.0134 (11)
C5	0.0442 (16)	0.0217 (13)	0.0295 (14)	0.0074 (12)	0.0126 (12)	0.0064 (11)
C6	0.0383 (15)	0.0247 (14)	0.0377 (16)	0.0017 (12)	0.0117 (13)	0.0099 (12)
C7	0.0310 (14)	0.0309 (15)	0.0452 (17)	0.0062 (12)	0.0160 (13)	0.0175 (13)
C8	0.0297 (13)	0.0266 (13)	0.0314 (14)	0.0098 (11)	0.0145 (11)	0.0140 (11)
C9	0.0257 (12)	0.0235 (12)	0.0233 (12)	0.0085 (10)	0.0089 (10)	0.0129 (10)
C10	0.0241 (12)	0.0290 (13)	0.0254 (13)	0.0075 (10)	0.0115 (10)	0.0120 (11)
C11	0.0250 (12)	0.0209 (12)	0.0223 (12)	0.0075 (10)	0.0092 (10)	0.0058 (10)
C12	0.0261 (13)	0.0324 (14)	0.0308 (14)	0.0065 (11)	0.0097 (11)	0.0104 (12)
C13	0.0309 (13)	0.0190 (12)	0.0222 (12)	0.0080 (10)	0.0101 (10)	0.0047 (10)
C14	0.0316 (13)	0.0274 (14)	0.0229 (13)	0.0095 (11)	0.0054 (11)	0.0050 (11)
C15	0.0416 (15)	0.0335 (15)	0.0196 (13)	0.0139 (12)	0.0080 (11)	0.0096 (11)
C16	0.0428 (15)	0.0335 (15)	0.0253 (14)	0.0126 (12)	0.0167 (12)	0.0127 (12)
C17	0.0328 (13)	0.0280 (13)	0.0231 (13)	0.0090 (11)	0.0113 (11)	0.0061 (11)
C18	0.0272 (12)	0.0201 (12)	0.0183 (12)	0.0073 (10)	0.0084 (10)	0.0047 (10)
C19	0.0304 (13)	0.0337 (14)	0.0246 (13)	0.0112 (11)	0.0137 (11)	0.0143 (11)
C20	0.0232 (12)	0.0333 (14)	0.0235 (12)	0.0100 (11)	0.0085 (10)	0.0145 (11)
C21	0.0373 (15)	0.0484 (18)	0.0414 (17)	0.0171 (13)	0.0180 (13)	0.0324 (15)
C22	0.0299 (13)	0.0350 (15)	0.0248 (13)	0.0121 (12)	0.0058 (11)	0.0141 (12)
C23	0.0465 (17)	0.0358 (16)	0.0332 (15)	0.0196 (14)	0.0088 (13)	0.0179 (13)
C24	0.060 (2)	0.0393 (17)	0.0343 (16)	0.0317 (16)	0.0127 (15)	0.0139 (14)
C25	0.0554 (19)	0.0497 (18)	0.0280 (15)	0.0328 (16)	0.0175 (14)	0.0138 (14)
C26	0.0420 (15)	0.0389 (16)	0.0276 (14)	0.0224 (13)	0.0157 (12)	0.0153 (12)
C27	0.0288 (13)	0.0289 (13)	0.0233 (13)	0.0131 (11)	0.0065 (10)	0.0095 (11)
C28	0.0235 (13)	0.0390 (16)	0.0241 (14)	0.0085 (12)	0.0083 (11)	0.0120 (13)
C29	0.0312 (17)	0.037 (2)	0.0329 (17)	0.0101 (15)	0.0172 (14)	0.0168 (16)
C29'	0.039 (19)	0.08 (3)	0.09 (3)	0.04 (2)	0.04 (2)	0.06 (3)
C30	0.058 (2)	0.076 (3)	0.0445 (19)	0.0227 (19)	0.0338 (17)	0.0312 (19)
C31	0.159 (6)	0.052 (3)	0.113 (4)	0.012 (3)	0.100 (4)	0.023 (3)
C32	0.079 (3)	0.045 (2)	0.063 (2)	0.0224 (19)	0.033 (2)	0.0117 (18)
C33	0.061 (2)	0.047 (2)	0.064 (2)	0.0267 (18)	0.0360 (19)	0.0236 (18)
C34	0.065 (2)	0.094 (3)	0.070 (3)	0.049 (2)	0.049 (2)	0.045 (2)

### Geometric parameters (Å, °)

**Table:**

Co—O1'	1.772 (13)	C12—H12A	0.9800
Co—O1	1.988 (2)	C12—H12B	0.9800
Co—N1	2.053 (2)	C12—H12C	0.9800
Co—N3	2.054 (2)	C13—C14	1.393 (4)
Co—N5	2.078 (2)	C13—C18	1.407 (4)
Co—N7	2.351 (2)	C14—C15	1.375 (4)
Cl—O3	1.415 (3)	C14—H14	0.9500
Cl—O4	1.416 (3)	C15—C16	1.408 (4)
Cl—O6	1.436 (2)	C15—H15	0.9500
Cl—O5	1.442 (3)	C16—C17	1.385 (4)
O1—C28	1.2697 (10)	C16—H16	0.9500
O2—C28	1.2403 (10)	C17—C18	1.390 (4)
O1'—C28	1.2697 (10)	C17—H17	0.9500
O2'—C28	1.2401 (10)	C19—C20	1.497 (4)
O7—C33	1.229 (5)	C19—H19A	0.9900
O8—C34	1.391 (4)	C19—H19B	0.9900
O8—H8O	0.8400	C21—H21A	0.9800
N1—C2	1.321 (3)	C21—H21B	0.9800
N1—C9	1.395 (3)	C21—H21C	0.9800
N2—C2	1.350 (3)	C22—C23	1.389 (4)
N2—C4	1.384 (3)	C22—C27	1.398 (4)
N2—C3	1.462 (3)	C23—C24	1.370 (5)
N3—C11	1.325 (3)	C23—H23	0.9500
N3—C18	1.402 (3)	C24—C25	1.390 (5)
N4—C11	1.344 (3)	C24—H24	0.9500
N4—C13	1.387 (4)	C25—C26	1.381 (4)
N4—C12	1.462 (3)	C25—H25	0.9500
N5—C20	1.327 (3)	C26—C27	1.385 (4)
N5—C27	1.400 (3)	C26—H26	0.9500
N6—C20	1.345 (3)	C28—C29'	1.27 (4)
N6—C22	1.382 (4)	C28—C29	1.488 (4)
N6—C21	1.466 (4)	C29—C30	1.297 (5)
N7—C10	1.470 (3)	C29—H29	0.9500
N7—C19	1.470 (3)	C29'—C30	1.31 (4)
N7—C1	1.476 (3)	C29'—H29'	0.9500
N10—C33	1.318 (5)	C30—H30A	0.9500
N10—C32	1.443 (5)	C30—H30B	0.9500
N10—C31	1.461 (6)	C31—H31A	0.9800
C1—C2	1.486 (3)	C31—H31B	0.9800
C1—H1A	0.9900	C31—H31C	0.9800
C1—H1B	0.9900	C32—H32A	0.9800
C3—H3A	0.9800	C32—H32B	0.9800
C3—H3B	0.9800	C32—H32C	0.9800
C3—H3C	0.9800	C33—H33	0.9500
C4—C5	1.395 (4)	C34—H34A	0.9800
C4—C9	1.401 (4)	C34—H34B	0.9800
C5—C6	1.372 (4)	C34—H34C	0.9800
C5—H5	0.9500	C35—O9	1.441 (3)
C6—C7	1.403 (4)	C35—H35A	0.9800
C6—H6	0.9500	C35—H35B	0.9800
C7—C8	1.387 (4)	C35—H35C	0.9800
C7—H7	0.9500	O9—H9O	0.8400
C8—C9	1.391 (4)	O9'—C35'	1.439 (3)
C8—H8	0.9500	O9'—H9O'	0.8400
C10—C11	1.484 (4)	C35'—H35D	0.9800
C10—H10A	0.9900	C35'—H35E	0.9800
C10—H10B	0.9900	C35'—H35F	0.9800
			
O1'—Co—O1	34.4 (6)	C14—C15—H15	119.2
O1'—Co—N1	123.9 (7)	C16—C15—H15	119.2
O1—Co—N1	91.92 (8)	C17—C16—C15	121.6 (3)
O1'—Co—N3	101.8 (7)	C17—C16—H16	119.2
O1—Co—N3	108.26 (9)	C15—C16—H16	119.2
N1—Co—N3	114.07 (8)	C16—C17—C18	117.4 (3)
O1'—Co—N5	87.5 (5)	C16—C17—H17	121.3
O1—Co—N5	113.55 (9)	C18—C17—H17	121.3
N1—Co—N5	112.69 (8)	C17—C18—N3	131.4 (2)
N3—Co—N5	114.27 (8)	C17—C18—C13	120.3 (2)
O1'—Co—N7	158.7 (6)	N3—C18—C13	108.3 (2)
O1—Co—N7	166.76 (8)	N7—C19—C20	106.8 (2)
N1—Co—N7	75.20 (8)	N7—C19—H19A	110.4
N3—Co—N7	75.19 (8)	C20—C19—H19A	110.4
N5—Co—N7	75.09 (8)	N7—C19—H19B	110.4
O3—Cl—O4	110.2 (2)	C20—C19—H19B	110.4
O3—Cl—O6	109.93 (17)	H19A—C19—H19B	108.6
O4—Cl—O6	111.49 (18)	N5—C20—N6	112.5 (2)
O3—Cl—O5	108.3 (2)	N5—C20—C19	121.7 (2)
O4—Cl—O5	108.69 (18)	N6—C20—C19	125.7 (2)
O6—Cl—O5	108.10 (14)	N6—C21—H21A	109.5
C28—O1—Co	121.23 (17)	N6—C21—H21B	109.5
C28—O1'—Co	139.7 (14)	H21A—C21—H21B	109.5
C34—O8—H8O	109.5	N6—C21—H21C	109.5
C2—N1—C9	105.8 (2)	H21A—C21—H21C	109.5
C2—N1—Co	117.75 (17)	H21B—C21—H21C	109.5
C9—N1—Co	136.15 (17)	N6—C22—C23	131.5 (3)
C2—N2—C4	107.0 (2)	N6—C22—C27	106.3 (2)
C2—N2—C3	127.1 (2)	C23—C22—C27	122.1 (3)
C4—N2—C3	125.9 (2)	C24—C23—C22	116.4 (3)
C11—N3—C18	105.5 (2)	C24—C23—H23	121.8
C11—N3—Co	117.02 (16)	C22—C23—H23	121.8
C18—N3—Co	136.71 (17)	C23—C24—C25	122.1 (3)
C11—N4—C13	107.2 (2)	C23—C24—H24	118.9
C11—N4—C12	126.3 (2)	C25—C24—H24	118.9
C13—N4—C12	126.5 (2)	C26—C25—C24	121.5 (3)
C20—N5—C27	105.8 (2)	C26—C25—H25	119.3
C20—N5—Co	117.45 (17)	C24—C25—H25	119.3
C27—N5—Co	136.56 (18)	C25—C26—C27	117.3 (3)
C20—N6—C22	107.2 (2)	C25—C26—H26	121.3
C20—N6—C21	127.4 (2)	C27—C26—H26	121.3
C22—N6—C21	125.3 (2)	C26—C27—C22	120.4 (3)
C10—N7—C19	111.3 (2)	C26—C27—N5	131.4 (3)
C10—N7—C1	112.0 (2)	C22—C27—N5	108.1 (2)
C19—N7—C1	112.4 (2)	O2'—C28—O2	144.7 (14)
C10—N7—Co	107.01 (15)	O2'—C28—C29'	111 (3)
C19—N7—Co	107.62 (15)	O2—C28—C29'	85 (3)
C1—N7—Co	106.10 (15)	O2'—C28—O1'	103 (2)
C33—N10—C32	121.7 (3)	O2—C28—O1'	71.9 (11)
C33—N10—C31	121.1 (3)	C29'—C28—O1'	146 (3)
C32—N10—C31	117.2 (4)	O2'—C28—O1	54.9 (17)
N7—C1—C2	107.9 (2)	O2—C28—O1	123.8 (2)
N7—C1—H1A	110.1	C29'—C28—O1	148 (2)
C2—C1—H1A	110.1	O1'—C28—O1	52.9 (10)
N7—C1—H1B	110.1	O2'—C28—C29	74.2 (17)
C2—C1—H1B	110.1	O2—C28—C29	120.1 (2)
H1A—C1—H1B	108.4	C29'—C28—C29	37 (3)
N1—C2—N2	112.7 (2)	O1'—C28—C29	163.6 (11)
N1—C2—C1	121.5 (2)	O1—C28—C29	116.1 (2)
N2—C2—C1	125.7 (2)	C30—C29—C28	123.5 (4)
N2—C3—H3A	109.5	C30—C29—H29	118.2
N2—C3—H3B	109.5	C28—C29—H29	118.2
H3A—C3—H3B	109.5	C28—C29'—C30	145 (4)
N2—C3—H3C	109.5	C28—C29'—H29'	107.4
H3A—C3—H3C	109.5	C30—C29'—H29'	107.4
H3B—C3—H3C	109.5	C29—C30—C29'	41 (3)
N2—C4—C5	132.2 (3)	C29—C30—H30A	120.0
N2—C4—C9	106.0 (2)	C29'—C30—H30A	81.9
C5—C4—C9	121.8 (3)	C29—C30—H30B	120.0
C6—C5—C4	116.9 (3)	C29'—C30—H30B	153.8
C6—C5—H5	121.6	H30A—C30—H30B	120.0
C4—C5—H5	121.6	N10—C31—H31A	109.5
C5—C6—C7	122.0 (3)	N10—C31—H31B	109.5
C5—C6—H6	119.0	H31A—C31—H31B	109.5
C7—C6—H6	119.0	N10—C31—H31C	109.5
C8—C7—C6	121.1 (3)	H31A—C31—H31C	109.5
C8—C7—H7	119.5	H31B—C31—H31C	109.5
C6—C7—H7	119.5	N10—C32—H32A	109.5
C7—C8—C9	117.5 (3)	N10—C32—H32B	109.5
C7—C8—H8	121.3	H32A—C32—H32B	109.5
C9—C8—H8	121.3	N10—C32—H32C	109.5
C8—C9—N1	130.8 (2)	H32A—C32—H32C	109.5
C8—C9—C4	120.8 (2)	H32B—C32—H32C	109.5
N1—C9—C4	108.4 (2)	O7—C33—N10	125.8 (4)
N7—C10—C11	108.0 (2)	O7—C33—H33	117.1
N7—C10—H10A	110.1	N10—C33—H33	117.1
C11—C10—H10A	110.1	O8—C34—H34A	109.5
N7—C10—H10B	110.1	O8—C34—H34B	109.5
C11—C10—H10B	110.1	H34A—C34—H34B	109.5
H10A—C10—H10B	108.4	O8—C34—H34C	109.5
N3—C11—N4	113.1 (2)	H34A—C34—H34C	109.5
N3—C11—C10	122.5 (2)	H34B—C34—H34C	109.5
N4—C11—C10	124.3 (2)	O9—C35—H35A	109.5
N4—C12—H12A	109.5	O9—C35—H35B	109.5
N4—C12—H12B	109.5	H35A—C35—H35B	109.5
H12A—C12—H12B	109.5	O9—C35—H35C	109.5
N4—C12—H12C	109.5	H35A—C35—H35C	109.5
H12A—C12—H12C	109.5	H35B—C35—H35C	109.5
H12B—C12—H12C	109.5	C35—O9—H9O	109.5
N4—C13—C14	131.7 (2)	C35'—O9'—H9O'	109.5
N4—C13—C18	105.9 (2)	O9'—C35'—H35D	109.5
C14—C13—C18	122.4 (3)	O9'—C35'—H35E	109.5
C15—C14—C13	116.7 (3)	H35D—C35'—H35E	109.5
C15—C14—H14	121.7	O9'—C35'—H35F	109.5
C13—C14—H14	121.7	H35D—C35'—H35F	109.5
C14—C15—C16	121.6 (2)	H35E—C35'—H35F	109.5
			
O1'—Co—O1—C28	24.9 (11)	C1—N7—C10—C11	84.9 (2)
N1—Co—O1—C28	−175.5 (2)	Co—N7—C10—C11	−31.0 (2)
N3—Co—O1—C28	−59.3 (2)	C18—N3—C11—N4	1.0 (3)
N5—Co—O1—C28	68.7 (2)	Co—N3—C11—N4	−170.57 (16)
N7—Co—O1—C28	−162.4 (3)	C18—N3—C11—C10	178.6 (2)
O1—Co—O1'—C28	−33.8 (17)	Co—N3—C11—C10	7.1 (3)
N1—Co—O1'—C28	−59 (3)	C13—N4—C11—N3	−1.2 (3)
N3—Co—O1'—C28	71 (3)	C12—N4—C11—N3	−178.7 (2)
N5—Co—O1'—C28	−174 (2)	C13—N4—C11—C10	−178.8 (2)
N7—Co—O1'—C28	150.8 (11)	C12—N4—C11—C10	3.7 (4)
O1'—Co—N1—C2	173.3 (7)	N7—C10—C11—N3	19.1 (3)
O1—Co—N1—C2	159.57 (19)	N7—C10—C11—N4	−163.5 (2)
N3—Co—N1—C2	48.6 (2)	C11—N4—C13—C14	179.7 (3)
N5—Co—N1—C2	−83.86 (19)	C12—N4—C13—C14	−2.8 (4)
N7—Co—N1—C2	−17.37 (17)	C11—N4—C13—C18	0.8 (3)
O1'—Co—N1—C9	0.4 (7)	C12—N4—C13—C18	178.3 (2)
O1—Co—N1—C9	−13.3 (2)	N4—C13—C14—C15	−179.9 (3)
N3—Co—N1—C9	−124.3 (2)	C18—C13—C14—C15	−1.2 (4)
N5—Co—N1—C9	103.3 (2)	C13—C14—C15—C16	0.6 (4)
N7—Co—N1—C9	169.8 (2)	C14—C15—C16—C17	0.3 (4)
O1'—Co—N3—C11	139.2 (6)	C15—C16—C17—C18	−0.6 (4)
O1—Co—N3—C11	174.25 (17)	C16—C17—C18—N3	−179.8 (3)
N1—Co—N3—C11	−85.03 (19)	C16—C17—C18—C13	0.0 (4)
N5—Co—N3—C11	46.6 (2)	C11—N3—C18—C17	179.4 (3)
N7—Co—N3—C11	−19.08 (17)	Co—N3—C18—C17	−11.6 (4)
O1'—Co—N3—C18	−28.9 (6)	C11—N3—C18—C13	−0.4 (3)
O1—Co—N3—C18	6.1 (3)	Co—N3—C18—C13	168.59 (18)
N1—Co—N3—C18	106.8 (2)	N4—C13—C18—C17	179.9 (2)
N5—Co—N3—C18	−121.5 (2)	C14—C13—C18—C17	0.9 (4)
N7—Co—N3—C18	172.8 (2)	N4—C13—C18—N3	−0.2 (3)
O1'—Co—N5—C20	179.8 (8)	C14—C13—C18—N3	−179.3 (2)
O1—Co—N5—C20	156.76 (18)	C10—N7—C19—C20	82.3 (2)
N1—Co—N5—C20	53.9 (2)	C1—N7—C19—C20	−151.1 (2)
N3—Co—N5—C20	−78.4 (2)	Co—N7—C19—C20	−34.6 (2)
N7—Co—N5—C20	−12.61 (18)	C27—N5—C20—N6	0.0 (3)
O1'—Co—N5—C27	−6.6 (8)	Co—N5—C20—N6	175.43 (16)
O1—Co—N5—C27	−29.6 (3)	C27—N5—C20—C19	−179.9 (2)
N1—Co—N5—C27	−132.4 (2)	Co—N5—C20—C19	−4.5 (3)
N3—Co—N5—C27	95.2 (2)	C22—N6—C20—N5	−0.9 (3)
N7—Co—N5—C27	161.0 (3)	C21—N6—C20—N5	178.5 (2)
O1'—Co—N7—C10	−56.6 (18)	C22—N6—C20—C19	179.0 (2)
O1—Co—N7—C10	134.8 (3)	C21—N6—C20—C19	−1.5 (4)
N1—Co—N7—C10	148.29 (17)	N7—C19—C20—N5	28.7 (3)
N3—Co—N7—C10	27.88 (15)	N7—C19—C20—N6	−151.2 (2)
N5—Co—N7—C10	−92.81 (16)	C20—N6—C22—C23	−177.3 (3)
O1'—Co—N7—C19	63.2 (18)	C21—N6—C22—C23	3.3 (5)
O1—Co—N7—C19	−105.4 (4)	C20—N6—C22—C27	1.4 (3)
N1—Co—N7—C19	−91.96 (16)	C21—N6—C22—C27	−178.0 (2)
N3—Co—N7—C19	147.62 (17)	N6—C22—C23—C24	−179.1 (3)
N5—Co—N7—C19	26.94 (16)	C27—C22—C23—C24	2.3 (4)
O1'—Co—N7—C1	−176.3 (18)	C22—C23—C24—C25	−0.1 (5)
O1—Co—N7—C1	15.1 (4)	C23—C24—C25—C26	−1.7 (5)
N1—Co—N7—C1	28.55 (15)	C24—C25—C26—C27	1.1 (4)
N3—Co—N7—C1	−91.87 (16)	C25—C26—C27—C22	1.1 (4)
N5—Co—N7—C1	147.44 (16)	C25—C26—C27—N5	−179.3 (3)
C10—N7—C1—C2	−150.4 (2)	N6—C22—C27—C26	178.2 (2)
C19—N7—C1—C2	83.4 (3)	C23—C22—C27—C26	−2.9 (4)
Co—N7—C1—C2	−34.0 (2)	N6—C22—C27—N5	−1.5 (3)
C9—N1—C2—N2	−0.4 (3)	C23—C22—C27—N5	177.4 (2)
Co—N1—C2—N2	−175.23 (16)	C20—N5—C27—C26	−178.7 (3)
C9—N1—C2—C1	177.3 (2)	Co—N5—C27—C26	7.2 (5)
Co—N1—C2—C1	2.5 (3)	C20—N5—C27—C22	0.9 (3)
C4—N2—C2—N1	0.2 (3)	Co—N5—C27—C22	−173.20 (19)
C3—N2—C2—N1	177.5 (2)	Co—O1'—C28—O2'	13 (3)
C4—N2—C2—C1	−177.4 (2)	Co—O1'—C28—O2	−131 (3)
C3—N2—C2—C1	−0.1 (4)	Co—O1'—C28—C29'	−180 (4)
N7—C1—C2—N1	24.3 (3)	Co—O1'—C28—O1	38.1 (18)
N7—C1—C2—N2	−158.3 (2)	Co—O1'—C28—C29	90 (4)
C2—N2—C4—C5	−179.8 (3)	Co—O1—C28—O2'	125.3 (17)
C3—N2—C4—C5	2.7 (4)	Co—O1—C28—O2	−11.5 (4)
C2—N2—C4—C9	0.2 (3)	Co—O1—C28—C29'	−163 (4)
C3—N2—C4—C9	−177.3 (2)	Co—O1—C28—O1'	−24.6 (12)
N2—C4—C5—C6	179.7 (3)	Co—O1—C28—C29	169.7 (2)
C9—C4—C5—C6	−0.3 (4)	O2'—C28—C29—C30	−140.1 (15)
C4—C5—C6—C7	0.5 (4)	O2—C28—C29—C30	4.6 (5)
C5—C6—C7—C8	−0.6 (5)	C29'—C28—C29—C30	26 (3)
C6—C7—C8—C9	0.4 (4)	O1'—C28—C29—C30	139 (3)
C7—C8—C9—N1	−179.5 (3)	O1—C28—C29—C30	−176.6 (3)
C7—C8—C9—C4	−0.2 (4)	O2'—C28—C29'—C30	−26 (8)
C2—N1—C9—C8	179.8 (3)	O2—C28—C29'—C30	121 (7)
Co—N1—C9—C8	−6.8 (4)	O1'—C28—C29'—C30	168 (5)
C2—N1—C9—C4	0.5 (3)	O1—C28—C29'—C30	−82 (7)
Co—N1—C9—C4	173.88 (18)	C29—C28—C29'—C30	−40 (6)
N2—C4—C9—C8	−179.8 (2)	C28—C29—C30—C29'	−24 (2)
C5—C4—C9—C8	0.2 (4)	C28—C29'—C30—C29	43 (6)
N2—C4—C9—N1	−0.4 (3)	C32—N10—C33—O7	−2.0 (6)
C5—C4—C9—N1	179.6 (2)	C31—N10—C33—O7	175.7 (5)
C19—N7—C10—C11	−148.3 (2)		

### Hydrogen-bond geometry (Å, °)

**Table:**

*D*—H···*A*	*D*—H	H···*A*	*D*···*A*	*D*—H···*A*
O8—H8O···O7^i^	0.84	1.94	2.765 (4)	169

Symmetry codes: (i) −*x*+2, −*y*+2, −*z*+1.
